# Factors associated with developing a fear of falling in subjects with primary open-angle glaucoma

**DOI:** 10.1186/s12886-018-0706-5

**Published:** 2018-02-13

**Authors:** Sayaka Adachi, Kenya Yuki, Sachiko Awano-Tanabe, Takeshi Ono, Daisuke Shiba, Hiroshi Murata, Ryo Asaoka, Kazuo Tsubota

**Affiliations:** 1 Department of Ophthalmology, Keio University School of Medicine, Shinanomachi 35, Shinjuku-ku, Tokyo, Japan; 2 Department of Ophthalmology, the University of Tokyo, Graduate School of Medicine, 7-3-1 Hongo, Bunkyo-ku, Tokyo, Japan

**Keywords:** Fear of falling, Primary open-angle glaucoma, Visual field, Quality of life, Risk factor

## Abstract

**Background:**

To investigate the relationship between clinical risk factors, including visual field (VF) defects and visual acuity, and a fear of falling, among patients with primary open-angle glaucoma (POAG).

**Methods:**

All participants answered the following question at a baseline ophthalmic examination: Are you afraid of falling? The same question was then answered every 12 months for 3 years. A binocular integrated visual field was calculated by merging a patient’s monocular Humphrey field analyzer VFs, using the ‘best sensitivity’ method. The means of total deviation values in the whole, superior peripheral, superior central, inferior central, and inferior peripheral VFs were calculated. The relationship between these mean VF measurements, and various clinical factors, against patients’ baseline fear of falling and future fear of falling was analyzed using multiple logistic regression.

**Results:**

Among 392 POAG subjects, 342 patients (87.2%) responded to the fear of falling question at least twice in the 3 years study period. The optimal regression model for patients’ baseline fear of falling included age, gender, mean of total deviation values in the inferior peripheral VF and number of previous falls. The optimal regression equation for future fear of falling included age, gender, mean of total deviation values in the inferior peripheral VF and number of previous falls.

**Conclusion:**

Defects in the inferior peripheral VF area are significantly related to the development of a fear of falling.

## Background

Fear of falling can cause individuals to avoid particular activities in their daily life, even if they are physically able to carry out these actions [1]. Previous studies have suggested that fear of falling is associated with self-imposed restrictions on activity [2, 3], depression [4], reduced mobility [5], an increased risk of actual falling [6, 7], and reduced health-related quality of life (QOL) [5]. Fear of falling may be a consequence of a previous experience of falling and could be considered as “post-fall syndrome” [7]. On the other hand, fear is common in elderly individuals who have never experienced a fall [1, 8]. This suggests that additional factors, other than previous experience of falling, may be related to developing a fear of falling.

Glaucoma, the second leading cause of blindness in the world, affects approximately 64.3 million adults globally [9]. In glaucomatous optic neuropathy, retinal ganglion cells are slowly and progressively destroyed, with a concomitant loss of peripheral and central vision. Fear of falling is more prevalent in individuals with glaucoma than in those without a visual field (VF) defect [β = − 1.20 logits; 95% confidence interval (CI), − 1.87 to − 0.53; *P* < 0.001] [10]. Wang et al. reported that subjects with glaucoma were 2.8 times more likely to be inactive in their daily life due to a fear of falling, avoiding activities such as traveling out of town, walking to the neighborhood or even moving around their home [3]. We previously reported that subjects with severe POAG (MD value less than − 12 dB in the better eye) were 4 times more likely to have a fear of falling [11]. However, previous studies have analyzed the association between visual function and fear of falling in a cross sectional manner. Thus, it is of significant interest to investigate the relationship between visual function and the development of fear of falling in a longitudinal manner. A prospective study may demonstrate that worsening of visual field (VF) defects leads to an increased fear of falling. The current study investigates the longitudinal relationship between various risk factors, including previous experience of falling, visual field (VF) defects and visual acuity, against fear of falling among patients with POAG.

## Subjects and methods

This study’s procedures conformed to the tenets of the Declaration of Helsinki and to national (Japanese) and institutional (Keio University School of Medicine) regulations. The study was approved by the Ethics Committee of the Keio University School of Medicine (#2010293). All study subjects gave a written informed consent prior to being enrolled.

## Study design and subject enrolment

This was a prospective observational study. All patients between 40 and 85 years of age who visited Keio University Hospital (Tokyo, Japan), the Iidabashi Eye Clinic (Tokyo, Japan), or the Tanabe Eye Clinic (Yamanashi, Japan) between the period of May 1, 2011 and November 30, 2011 were screened for eligibility for this study.

### Baseline evaluation of subjects with glaucoma

Patients with glaucoma were consecutively screened for eligibility using a battery of ophthalmic examinations, including slit-lamp biomicroscopy, funduscopy, gonioscopy, intraocular pressure measurements by Goldmann applanation tonometry, and VF examination with the Humphrey field analyser (HFA) and the 24–2 Swedish Interactive Threshold Algorithm Standard Strategy (Carl Zeiss Meditec, Dublin, CA). The findings were analysed by T.S., and K.Y., both of whom subspecialize in glaucoma. The reliability of the visual field was confirmed to be sufficiently high, with less than a 20% fixation loss rate and less than a 15% false-positive rate [12].

### Diagnostic criteria for POAG

POAG was diagnosed according to the presence of the following three findings: (1) glaucomatous optic disc cupping, represented by notch formation, generalized cup enlargement, a senile sclerotic or myopic disc, or nerve-fibre layer defects; (2) glaucomatous VF defects, defined according to Anderson and Patella’s criteria (a cluster of 3 or more points in the pattern deviation plot within a single hemifield [superior or inferior] with a *p* value < 5%, one of which must have a p value < 1% [13]; and (3) an open angle observed on gonioscopy.

### Exclusion criteria

Subjects were excluded if they had an ophthalmologic disease other than POAG that could potentially compromise visual acuity or contribute to VF loss and senile cataract. Subjects were also excluded if they had a decimal BCVA of less than 0.7, were unable to walk without assistance, walked with a cane, or had a mental disorder that affected their ability to understand the questionnaire regarding falls and fear of falling as detailed below. IOP was not used as an exclusion criterion so both POAG and normal tension glaucoma patients were included in the study.

### Baseline falls questionnaire

All study participants answered the following questionnaire at their baseline ophthalmic examination (note that questions were originally written in Japanese); responses are listed in parentheses:

1. Are you afraid of falling? (Not at all/Not much/Afraid/Very afraid).
2. How many times did you fall in the last year?
3. Have you experienced any injurious falls in the last year? (Yes/No).

The question “Are you afraid of falling?” was used in previous studies to provide a severity grading for fear of falling [14–16]. Demographic information were recorded for all subjects, including age, sex, height, weight, systemic hypertension (HT), diabetes mellitus (DM), and usage of sleeping aids or tranquilizers (sedative/sleeping aid).

### Follow-up falls questionnaire

All study participants answered the following question again every 12 months (± 1 month) after the baseline questionnaire, for a total of 3 years:

(1) Are you afraid of falling? (Not at all / Not much / Afraid / Very Afraid).

Subjects who answered the question at least two times over the 3 years of follow-up were analyzed. The highest severity response was used to summarise subjects’ “future fear of falling” over the follow-up period. For example, subjects who answered “Not at all” at their first follow-up, “Afraid” at their second follow-up and “Very afraid” at their final follow-up were defined as “Very afraid” of falling. This annual survey was carried out following a previous report [16]. The “baseline fear of falling (+)” group was defined as those who answered ‘Afraid’ or ‘Very afraid’ at baseline interview, whereas the “baseline fear of falling (−)” group was defined as those who answered ‘Not at all’ or ‘Not much’. Similarly, the “future fear of falling (+)” group was defined as those who answered ‘Afraid’ or ‘Very afraid’ at follow-up interview, whereas the “future fear of falling (−)” group was defined as those who answered ‘Not at all’ or ‘Not much’ at follow-up interview.

### Integrated binocular visual field

**Fig. 1:**
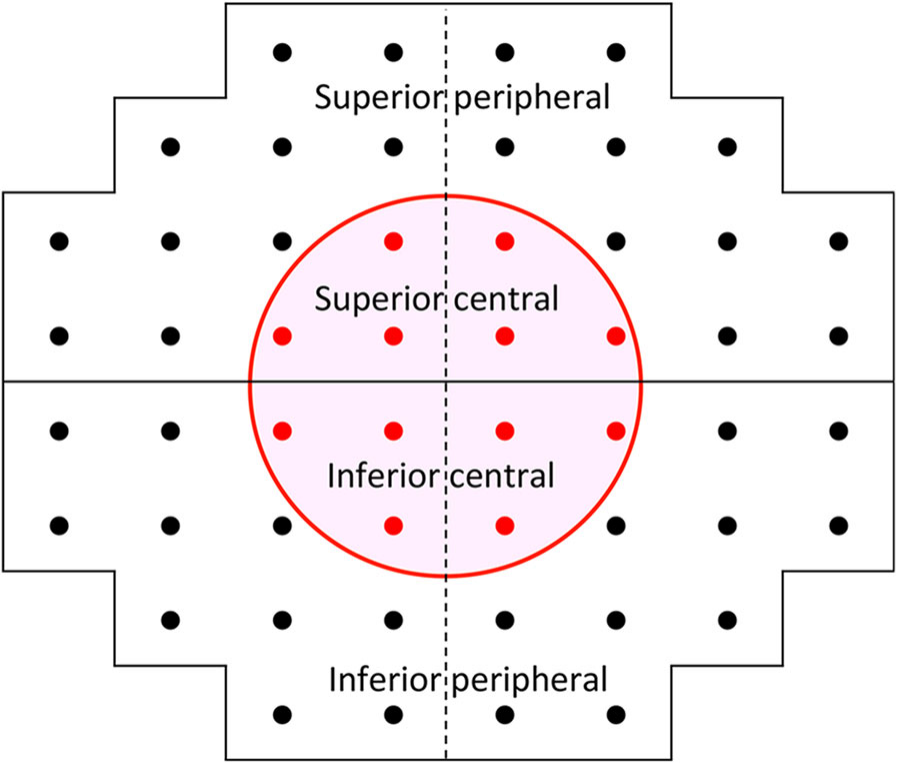
Mapping of the superior peripheral, superior central, inferior central and inferior peripheral areas. The VF was divided outside and within the central 10 degrees. These areas also follow mappings in the 24–2 and 10–2 visual fields of the Humphrey Field Analyzer

A binocular integrated visual field (IVF) was calculated for each patient by merging a patient’s monocular HFA VFs, using the ‘best sensitivity’ method, where the IVF total deviation (TD) at each point was calculated using the maximum TD (least negative) value from each of the two overlapping points, as if the subject was viewing the field binocularly [17]. The IVF MD was calculated as the mean of 52 TD values across the VF, while the means of TD values in the superior peripheral (mTD_sp_), superior central (mTD_sc)_, inferior central (mTD_IC_), and inferior peripheral (mTD_IP_) areas were also calculated, following the areas indicated in Fig. 1; thus, the VF was divided outside and within the central 10 degrees (these areas follow the mapping in the 24–2 and 10–2 visual field of the HFA).

### Statistical analysis

Descriptive statistics were calculated for the demographic, medical, and visual-function variables. These values were compared between the baseline fear of falling (+) and (−) group. Then the relationship between VF measurements, better-eye and worse-eye visual acuities and various clinical factors against baseline fear of fall was analyzed using the multiple logistic regression model. The optimal logistic model was selected among all possible combinations of predictors, which was 2 [13] combinations (predictors were: age, gender, better-eye visual acuity, worse-eye visual acuity, mTD_SP_, mTD_SC_, mTD_IC_, mTD_IP_, sedative/sleeping aid, BMI, HT, DM and the number of previous falls). The degrees of freedom in a multivariate logistic regression model decreases with a large number of variables so it is recommended to use model selection methods to improve the model fit by removing redundant variables [18, 19]. The test statistic used here to measure model fit was the second order bias corrected Akaike Information Criterion (AICc) index. The AIC is a common statistical measure used in model selection, and the AICc is a corrected version of the AIC, which gives an accurate estimation even when the sample size is small [20].

A comparison of demographic data was carried out between the future fear of falling (+) and (−) groups. Subsequently, the relationship between VF measurements, better-eye and worse-eye visual acuities and various clinical factors against baseline fear of fall was analyzed using the multiple logistic regression model.

The Chi square test was used to compare categorical data between the two groups, whereas the Wilcoxon rank-sum test was used to compare numerical data between the two groups. All analyses were performed using the statistical programming language ‘R’ (R version 2.15.1; The Foundation for Statistical Computing, Vienna, Austria).

## Results

Of the 556 POAG patients screened, 164 were excluded. The reasons for excluding subjects were as follows (numbers in parentheses indicate the number of subjects excluded): younger than 40 years old [28], older than 85 years old [25], refusal to participate [10], unable to walk without assistance (0), walked with a cane [12], dementia [3], low visual acuity [24], post retinal-detachment [21], diabetic retinopathy (36), bullous keratopathy [2], age-related macular degeneration [2], other ocular disease [1]. As a result, 392 POAG patients were eligible for the study. Among the 392 POAG eligible subjects, 342 patients (87.2%) answered the falls question at least twice over the 3 years study period and are analyzed here. Subject demographics are shown in Table 1.

**Table 1:** Demographic characteristics of subjects with POAG analyzed in this study.

	Mean	Standard deviation	Range
Number	342		
Age (years)	65.1	10.7	40 to 85
Gender (Male/Female)	197/145 (57.6 / 42.4%)		
B.M.I (kg/mm^2^)	22.5	3.1	13.8 to 32.7
Prevalence of diabetes mellitus (Yes)	49 (14.3%)		
Prevalence of systemic hypertension (Yes)	103 (30.1%)		
Better BCVA (LogMar)	0.004	0.02	0.15 to 0.0
Worse BCVA (LogMar)	0.018	0.04	0.15 to 0.0
mTD (dB)	−2.08	4.02	−20.6 to 5.3
mTD_SP_ (dB)	−2.83	5.59	−28.4 to 6.0
mTD_SC_ (dB)	−2.62	5.80	−32.3 to 5.3
mTD_IC_ (dB)	−0.89	3.35	−29.2 to 6.2
mTD_ip_ (dB)	−1.54	3.52	−20.9 to 4.3
Use of sedative/sleeping aid (Yes)	21 (6.1%)		
Number of previous fall (0/1/2 or more)	288/30/24 (84.2%/8.8%/7.0%)		
Previous history of injurious fall (Yes/No)	21 (6.1%)		

*Abbreviations*: *POAG*, primary open-angle glaucoma, *B.M.I*, body mass index, *LogMar*, the logarithm of the minimum angle, *BCVA*, best corrected visual acuity, *mTD*, mean total deviation, *SP*, superior peripheral, *SC*, superior central, *IC*, inferior central, *IP*, inferior peripheral

Among 342 patients, 43 patients were categorized in the baseline fear of falling (+) group. The comparison of systemic and ocular demographic characteristics between the baseline fear of falling (−) group and the baseline fear of falling (+) group are shown in Table 2. Age, BCVA in the better eye, BCVA in the worse eye, mTD, mTD_SP_, mTD_SC_, mTD_IC_, mTD_IP_, past history of falls and the number of previous falls were significantly different between the two groups (Wilcoxon test or chisquare test, *p* < 0.05). As shown in Table 3, the optimal model for patients’ baseline fear of falling was; baseline fear of falling (+) = − 7.0 + 0.062 * age + 0.58 * female – 0.088 * mTD_IP_ + 0.87 * number of previous falls (AICc = 224.5, R^2^ = 0.17), where ‘female’ was assigned a value equal to 1 and male a value equal to 0.

**Table 2:** Comparison of visual and systemic parameters between baseline fear of falling + group and baseline fear of falling – group

	Baseline fear of falling (−)	Baseline fear of falling (+)	*p value*
Number	299	43	
Age (years)	64.3 ± 10.6 [45 to 85]	70.9 ± 9.8 [40 to 84]	< 0.0001
Gender (male/female)	178/121	19/24	0.082
B.M.I	22.5 ± 3.0 [13.8 to 32.7]	22.1 ± 3.6 [15.2 to 32.0]	0.47
Prevalence of diabetes mellitus (%)	40/299 = 13.4%	9/43 = 20.9%	0.28
Prevalence of hypertension (%)	92/299 = 30.8%	11/43 = 25.6%	0.61
Usage of sedatives/sleeping aid (%)	15/299 = 5.0%	6/43 = 13.6%	0.052
BCVA in the better eye (LogMar)	0.003 ± 0.015 [0.0 to 0.15]	0.011 ± 0.034 [0.0 to 0.15]	0.003
BCVA in the worse eye (LogMar)	0.015 ± 0.038 [0.0 to 0.15]	0.037 ± 0.056 [0.0 to 0.15]	0.001
mTD (dB)	−3.8 ± 4.9 [−20.3 to 5.3]	−2.0 ± 3.9 [−20.6 to 3.2]	0.0038
mTD_SP_ (dB)	−4.8 ± 7.1 [−28.2 to 6.0]	− 2.7 ± 5.4 [− 28.4 to 3.9]	0.014
mTD_SC_ (dB)	−5.0 ± 7.8 [− 32.3 to 5.3]	−2.5 ± 5.6 [− 32.3 to 3.3]	0.0094
mTD_IC_ (dB)	− 2.1 ± 4.1 [−18.0 to 6.2]	−0.83 ± 3.3 [− 29.2 to 3.3]	0.0021
mTD_ip_ (dB)	−2.9 ± 4.1 [− 20.9 to 4.3]	−1.5 ± 3.4 [− 20.6 to 3.6]	0.0049
Past history of falls (%)	37/299 = 12.4%	18/43 = 41.9%	< 0.0001
Number of previous fall (times)	0.18 ± 0.54 [0 to 6]	0.89 ± 4.1 [0 to 4]	< 0.0001

The Chi square test was used to compare categorical data between the two groups, whereas the Wilcoxon test was used to compare numerical data between the two groups

*Abbreviations*: *B.M.I*, body mass index, *BCVA*, best corrected visual acuity, *LogMar*, logarithm of the Minimum Angle of Resolution, *mTD*, mean total deviation, *SP*, superior peripheral, *SC*, superior central, *IC*, inferior central, *IP*, inferior peripheral

**Table 3:** The optimal model for baseline fear of falling

Parameters used in the model selection	Selected parameters’ coefficients
Age	0.062
Gender	0.58 (female)
Better visual acuity	NS
Worse visual acuity	NS
mTD_SP_	NS
mTD_SC_	NS
mTD_IC_	NS
mTD_IP_	−0.088
Sedative/sleeping aid	NS
BMI	NS
DM	NS
HT	NS
Number of previous fall	0.87

*Abbreviations*: *TD*, total deviation, *SP*, superior peripheral, *SC*, superior central, *IC*, inferior central, *IP*, inferior peripheral, *BMI*, body mass index, *DM*, diabetes mellitus, *HT*, hypertension, *NS* represents not selected in the optimal model, *mTD*, mean total deviation, *SP*, superior peripheral, *SC*, superior central, *IC*, inferior central, *IP*, inferior peripheral, *NS* represents not selected in the optimal model

Among 342 patients, 94 patients were categorized in the future fear of falling (+) group. The comparisons of systemic and ocular demographic characteristics are shown in Table 4. There was a significant difference in age, gender, BCVA in the better eye, BCVA in the worse eye, mTD, mTD_IC_, mTD_IP_, past history of falls and number of previous falls (Wilcoxon test or chisquare test, *p* < 0.05). As shown in Table 5, the optimal regression equation for future fear of falling was: future fear of falling (+) = − 6.2 + 0.068 * age + 0.77 * female - 0.058 * mTD_IP_ + 0.83 * number of previous falls (AICc = 350.1, R^2^ = 0.16), where ‘female’ and ‘male’ was assigned the value of 1 and 0, respectively.

**Table 4:** Comparison of visual and systemic parameters between the future fear of falling + group and the future fear of falling - group

Parameters at baseline	Future Fear of falling (−)	Future Fear of falling (+)	*p value*
Number	248	94	
Age (years)	63.2 ± 10.4	70.1 ± 9.8	< 0.0001
Gender (male/female)	155/93	42/52	0.026
B.M.I	22.5 ± 3.0	22.3 ± 3.2	0.56
Prevalence of diabetes mellitus (%)	35/213 = 14.1%	14/80 = 14.9%	0.63
Prevalence of hypertension (%)	76/172 = 30.6%	27/67 = 28.7%	0.76
Usage of sedative/sleeping aid	15/233 = 6.0%	6/88 = 6.4%	0.36
BCVA in the better eye (LogMar)	0.0021 ± 0.013	0.0093 ± 0.029	0.0021
BCVA in the worse eye (LogMar)	0.015 ± 0.038	0.028 ± 0.051	0.023
mTD (dB)	−2.0 ± 4.0	−2.9 ± 4.3	0.036
mTD_SP_ (dB)	−3.8 ± 6.3	− 2.7 ± 5.4	0.12
mTD_SC_ (dB)	− 2.6 ± 5.7	− 3.4 ± 6.7	0.17
mTD_IC_ (dB)	−1.3 ± 3.3	−0.86 ± 3.5	0.023
mTD_ip_ (dB)	−1.4 ± 3.5	− 2.3 ± 3.7	0.012
Past history of falls (%)	27/248 = 10.9%	28/94 = 29.8%	0.12
Number of previous fall	0.14 ± 0.46	0.60 ± 1.1	< 0.0001

The Chi square test was used to compare categorical data between two groups, whereas the Wilcoxon test was used to compare numerical data between the two groups

*Abbreviations*: *B.M.I*, body mass index, *BCVA*, best corrected visual acuity, *LogMar*, logarithm of the Minimum Angle of Resolution, *mTD*, mean total deviation, *SP*, superior peripheral, *SC*, superior central, *IC*, inferior central, *IP*, inferior peripheral

**Table 5:** The optimal model for future fear of falling

Parameters used in the model selection	Selected parameters’ coefficients
Age	0.068
Gender	0.77 (female)
Better visual acuity	NS
Worse visual acuity	NS
mTD_SP_	NS
mTD_SC_	NS
mTD_IC_	NS
mTD_IP_	−0.058
Sedative/sleeping aid	NS
BMI	NS
DM	NS
HT	NS
Number of previous fall	0.83

*Abbreviations*: *TD*, total deviation, *SP*, superior peripheral, *SC*, superior central, *IC*, inferior central, *IP*, inferior peripheral, *BMI*, body mass index, *DM*, diabetes mellitus, *HT*, hypertension, *mTD*, mean total deviation, *SP*, suprior peripheral, *SC*, superior central, *IC*, inferior central, *IP*, inferior peripheral. *NS* represents not selected in the optimal model

## Discussion

Our findings suggest that an inferior peripheral visual field defect is related to developing a fear of falling. Ramulu et al. performed a cross-sectional study and surveyed the severity of fear of falling in 83 control subjects with bilateral VF loss and 60 control subjects without VF defects; they found that fear of falling was more severe in subjects with glaucoma than in controls after multivariable adjustment (β = − 1.20 logits, *p* = 0.001) [10]. Wang et al. asked 98 glaucoma subjects (MD -9.7 ± 6.4 in the better eye) and 97 controls whether a fear of falling limited their activities; 42% of the subjects with glaucoma reported that fear of falling limited their activity, whereas the proportion was only 16% in the controls. After multivariable adjustment, the subjects with glaucoma were more likely than control subjects to report activity limitations due to a fear of falling (OR 2.84; 95% CI, 1.36–5.96) [3]. We have previously reported that the adjusted ORs for prevalence of fear of falling were 1.44 (95% CI: 0.83–2.51) in mild POAG, 2.33 (95% CI: 1.00–5.44) in moderate POAG, and 4.06 (95% CI: 1.39–11.90) in severe POAG, compared to controls [11].

None of these previous studies investigated the importance of the location of a VF defect on the fear of falling. This study suggests that deterioration of sensitivity in the inferior peripheral VF area is related to the development of fear of falling. Black et al. reported that damage in the inferior VF was associated with a higher rate of falls (RR, 1.57; 95% CI, 1.06 to 2.32) and falls with injury (RR, 1.80; 95% CI, 1.12 to 2.98) [21]. We previously examined the relationship between the area of a VF defect and the occurrence of a fall with injury in subjects with POAG. We found that damage to the lower peripheral VF was a significant risk factor for an injurious fall [22]. Inferior VF loss has also been shown to be associated with weaker lower limb strength and lower overall functional status scores in subjects with POAG [23]. Fear of falling is associated with increased risk of falls [7] and reduced physical function of lower limbs [23]. These studies strongly support our results that an inferior VF defect is related to developing a fear of falling in future.

In the Beaver Dam Eye study, the incidence of fear of falling was associated with the poorest category of habitual visual acuity (20/40 or worse) [OR, = 2.95 with a 95% CI of 1.52 to 5.70; the reference group had visual acuity of 20/20 or better] [24]. Ramulu et al. also reported that a severe fear of falling was associated with worse visual acuity in the better eye (β = − 0.14 logtis per 0.1 logMar increment; 95%CI = − 0.25 to − 0.03; *p* = 0.02) in subjects with glaucoma [10]. In the current study, visual acuities in the better and worse eyes were not included in the optimal model for fear of falling and instead inferior VF defect was included.

Our study also found that older age, frequent previous history of falls, and being female were significant risk factors for fear of falling. Older age and being female were also found to be significant risk factors for fear of falling in previous studies [7, 15, 25, 26].

Our study has several limitations. First, fear of falling was assessed using a single question that has not been validated, and falling was not precisely defined, “Are you afraid of falling?” This is likely to be less sensitive than other assessments, such as the Fall Efficacy Scale and Activities-Specific Balance Confidence Scale [27].. The use of a single question to determine a history of falls and the number of falls is not considered the gold-standard [28] so a future study should be conducted using a validated falls questionnaire. Second, all subjects in the study had glaucoma, and no controls were included. Since all subjects knew they had glaucoma and the disease is associated with anxiety [29], they may tend to pessimistically answer the question regarding fear of falling. Third, 50 patients were lost to follow up and we do not know the reason for this, which could bias the results. Fourth, the prevalence of a history of falls at baseline appears low in our study when compared with previous studies. The reason for this is unclear but one possible explanation is the age range of subjects in our study, which was relatively young (40 to 85 years) compared with earlier studies. Another possible cause is that falling risk, among older adults, varies by racial/ethnic groups. Kwon et al. reported that Asian Americans were significantly less likely to fall compared to non-Hispanic whites [30]. Geng et al. also reported that, compared to whites, Asian women were much less likely to have ≥1 fall in the past year (OR 0.64, CI 0.50–0.81), adjusting for age, comorbidities, mobility limitation and poor health status. Asians were also less likely to have ≥2 falls (OR 0.62, CI 0.43–0.88) [31].

## Conclusion

We found that a defect in the inferior VF area is significantly related to the development of a fear of falling. Halting the progression of VF defects, especially in the inferior VF, may reduce patients’ fear of falling and improve their health-related QOL.
